# A DNA Methylation Perspective on Infertility

**DOI:** 10.3390/genes14122132

**Published:** 2023-11-27

**Authors:** Ghaleb Shacfe, Rasoul Turko, Haadi Hammad Syed, Ibraheem Masoud, Yahya Tahmaz, Lara M. Samhan, Khaled Alkattan, Areez Shafqat, Ahmed Yaqinuddin

**Affiliations:** College of Medicine, Alfaisal University, Riyadh 11533, Saudi Arabia; gshacfe@alfaisal.edu (G.S.); aturko@alfaisal.edu (R.T.); hhsyed@alfaisal.edu (H.H.S.); imasoud@alfaisal.edu (I.M.); ytahmaz@alfaisal.edu (Y.T.); lsamhan@alfaisal.edu (L.M.S.); kkattan@alfaisal.edu (K.A.); ayaqinuddin@alfaisal.edu (A.Y.)

**Keywords:** epigenetics, infertility, DNA methylation, Artificial Reproductive Technology

## Abstract

Infertility affects a significant number of couples worldwide and its incidence is increasing. While assisted reproductive technologies (ART) have revolutionized the treatment landscape of infertility, a significant number of couples present with an idiopathic cause for their infertility, hindering effective management. Profiling the genome and transcriptome of infertile men and women has revealed abnormal gene expression. Epigenetic modifications, which comprise dynamic processes that can transduce environmental signals into gene expression changes, may explain these findings. Indeed, aberrant DNA methylation has been widely characterized as a cause of abnormal sperm and oocyte gene expression with potentially deleterious consequences on fertilization and pregnancy outcomes. This review aims to provide a concise overview of male and female infertility through the lens of DNA methylation alterations.

## 1. Introduction

Infertility can affect up to 8–12% of couples worldwide, and its incidence is increasing [1,2]. Around 7% of males are infertile and 1 in 20 men have sperm counts low enough to cause infertility [3]. Furthermore, sperm counts have declined precipitously over the past 40 years [4]. Female infertility is better studied compared to male, and approximately 6% of married women in the United States are infertile [5]. 

The most common listed causes of infertility in women are polycystic ovarian syndrome (PCOS), premature ovarian insufficiency, endometriosis, and hyperprolactinemia [6]. Varicocele is implicated as the most common cause of infertility in men [7]. Other factors like age, stress, lifestyle, genetic abnormalities, and infections of the genital tract can be associated with infertility in both genders [8]. However, often the cause remains unknown and, therefore, they are difficult to appropriately manage/treat. Assisted Reproductive Technology (ART), as per the definition by the Center for Disease Control and Prevention (CDC), includes any procedure that involves handling ova or embryos to aid fertilization/achieve pregnancy; procedures like intrauterine insemination or ovarian stimulation without oocyte retrieval are not considered a part of ART [9]. Well-known and frequently used ART procedures include in vitro fertilization (IVF), in vitro maturation, and intracytoplasmic sperm injection (ICSI) [9]. However, the prevalence of idiopathic infertility, which affects 15–30% of infertile couples [10], underscores the importance of identifying determinants of normal gametogenesis and embryogenesis. This is an area that requires more research and could prove to be essential in increasing the success rate of ART. 

Abnormal gene expression has been associated with both male and female infertility [7], and better studying the causes and biological implications of abnormal gene expression in germ cells may explain some of the aforementioned idiopathic cases. Epigenetics, including DNA methylation, histone post-translational modifications, and RNA interference, is a form of regulating gene expression without altering nucleotide sequence and is a dynamic process influenced by different biological and environmental factors [11]. Notably, specific epigenetic marks need to be established during different stages of gametogenesis to form morphologically and functionally mature sperm [12,13]. DNA methylation is the most widely studied form of epigenetic regulation and has been associated with reduced male and female reproductive potential and aberrant improper post-fertilization embryo development [14]. Epigenetic modifications like DNA methylation also contribute to a host of pregnancy-associated complications and fetal outcomes like growth restriction [15,16].

Therefore, studying specific genes affected by abnormal DNA methylation may explain idiopathic infertility cases; better the understanding of the precise regulators of gametogenesis and embryogenesis; improve the clinical assessment of couples affected by infertility; improve the detection of women at risk of adverse pregnancy outcomes or fetal complications; and reveal novel targets for therapy to treat such cases. To curate studies on DNA methylation and fertility, we conducted a literature search on PubMed, MEDLINE, and Google Scholar using the terms “Methylation”, “Male”, “Female”, “Fertility”, “Infertility”, and “Epigenetics” in various combinations. 

## 2. Overview of DNA Methylation 

**Figure 1:**
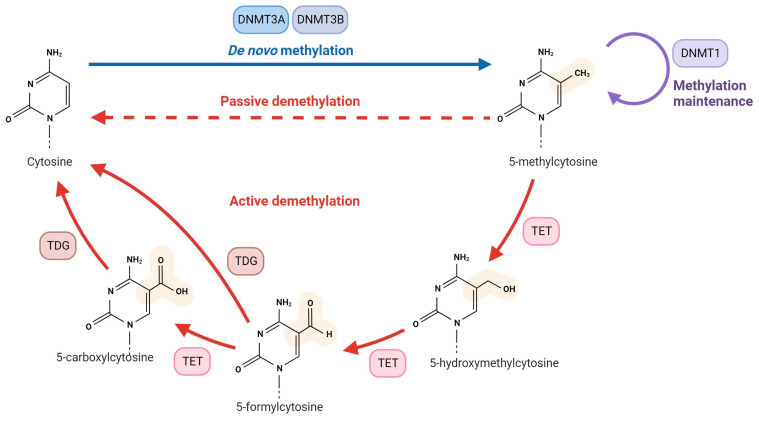
DNA methyltransferases (DNMTs) add a methyl group to the 5′ carbon position of a cytosine in a CpG context. DNMT1 copies pre-existing methylation marks onto daughter DNA strands after DNA replication, whereas DNMT3A and DNMT3B methylate previously unmethylated DNA sequences. TET1, -2, and -3 are responsible for demethylation by oxidizing 5-mC to 5-hydroxymethylcytosine (5-hmC), which is not recognized by DNMT1 and is hence lost during replication. Further oxidation of 5-hmC produces 5-formyl cytosine (5-fC) and 5-carboxyl cytosine (5-caC, which are recognized and excised by base-excision DNA repair machinery like thymine DNA glycosylases (TDGs) and replaced with cytosine. Reprinted from “DNA Methylation” template by BioRender.com. Retrieved from https://app.biorender.com/biorender-templates/figures.bio (accessed 3 November 2023).

DNA methylation is a process whereby DNA methyltransferases (DNMTs) add a methyl group to the 5′ carbon of a cytosine in a cytosine-guanine (CpG) dinucleotide context to form 5-methylcytosine (5-mC) (Figure 1) [7]. Methylation of regulatory regions such as promoters and enhancers contain CpG islands (CGI) interferes with transcription factor binding and results in gene silencing [7,17]. A second pathway of methylation-induced gene silencing involves methyl CpG binding domain (MBD) proteins, which recognize 5-mC and recruit chromatin modifiers that modify histones and affect chromatin packing to reinforce gene silencing [18]. Different types of DNMTs—namely, DNMT1, DNMT3A, DNMT3B, and DNMT3C—coordinate to establish physiological DNA methylation marks. DNMT1 copies pre-existing methylation marks onto daughter DNA strands after DNA replication, functioning thereby as a ‘maintenance DNMT’, whereas DNMT3A and DNMT3B methylate previously unmethylated DNA sequences (i.e., de novo DNMTs) [19]. Ten-Eleven Translocation (TET) enzymes TET1, -2, and -3 are responsible for demethylation by oxidizing 5-mC to 5-hydroxymethylcytosine (5-hmC), which is not recognized by DNMT1 and is hence lost during replication. Further oxidation of 5-hmC produces 5-formyl cytosine (5-fC) and 5-carboxyl cytosine (5-caC), which are recognized and excised by base-excision DNA repair machinery like thymine DNA glycosylase (TGD) enzymes and replaced with cytosine (19).

## 3. DNA Methylation during Embryogenesis 

**Figure 2:**
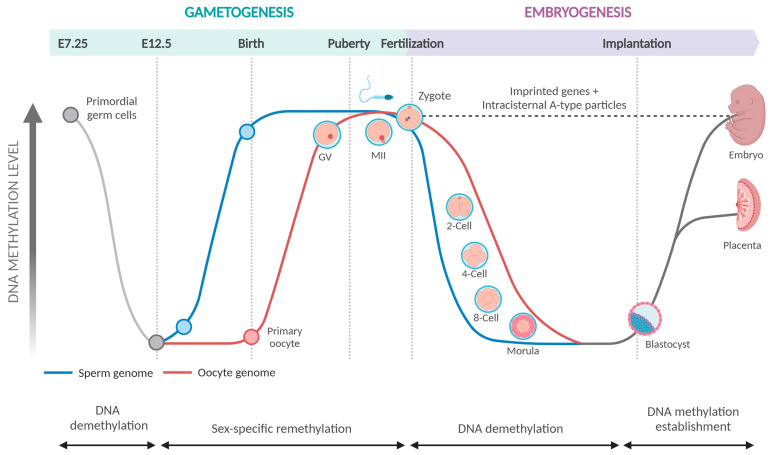
DNA methylation during mammalian development. The genome of embryonic cells initially experiences a wave of DNA demethylation. A second wave of DNA methylation affecting imprinted loci and germline-specific loci occurs in primordial germ cells PGCs, enabling the formation of sex-specific germ cells during embryogenesis. DNA methylation in oocytes and sperms differs significantly. Germ cells of the female embryo enter meiosis-I and are arrested in prophase 1, remaining inactive until embryos are born, whereas methylation of gDMRs in sperm beings in the fetal testis and is nearly complete by birth. Reprinted from “DNA Methylation during Mammalian Development” template on BioRender.com. Retrieved from https://app.biorender.com/biorender-templates/figures (accessed on 3 November 2023).

The genome of embryonic cells initially experiences a wave of DNA demethylation, conferring to these cells pluripotent potential for future lineage specification (Figure 2). Another function of removing DNA methylation marks may be to prevent transmission of acquired epimutations to offspring. This DNA methylation erasure could either be active (paternal genome) or passive (maternal genome). DNA methylation is also essential for normal germ-cell development [20,21]. A second wave of DNA methylation affecting imprinted loci and germline-specific loci occurs in primordial germ cells (PGCs) when they migrate from the epiblast to the gonadal ridge [22]. As a result, PGCs have lower DNA methylation levels than embryo somatic cells [23,24]. These epigenetic changes are necessary for the genomic reprogramming of PGCs, which enables the formation of sex-specific germ cells during embryogenesis. De novo methylation takes place in gonocytes or prospermatogonia that occur first at imprinted genes before moving onto repeated DNA sequences like retrotransposons. This results in sex-specific methylation patterns in the germ cells [7]. To guarantee correct embryonic growth and sperm function throughout subsequent spermatogenesis, proper epigenetic process regulation is essential. 

## 4. DNA Methylation during Gametogenesis 

A small number of cells are set aside as PGCs in early post-implantation embryos. These cells eventually mature into germ cells (sperm or oocytes) for reproduction. To produce germ cell-specific epigenomes, somatic lineage epigenetic markers, such as DNA methylation, are removed from PGCs during epigenetic reprogramming in the germline [25]. DNA methylation in oocytes and sperms differs significantly (Figure 2) [26]. Germ cells of the female embryo are arrested in prophase 1, remaining inactive until embryos are born. After birth, the oocytes expand to their full size and transition from the main follicle stage to the secondary follicle stage after entering the growth phase, which is when methylation at germline differentially methylated regions (gDMRs) is established [27,28]. On the other hand, in male germ cells, methylation of gDMRs occurs before the commencement of meiosis in prospermatogonia, beginning in the fetal testis and is nearly complete by birth [29]. There are multiple rounds of cell division between the start of de novo methylation and the production of mature sperm, so initially determined methylation patterns may be modified through maintenance, and there is a greater chance for methylation errors to accumulate. 

## 5. DNA Methylation and Male Infertility 

Most research on infertility has revolved around defining causes and devising interventions for female infertility. Approximately 30–50% of infertility cases can involve male infertility [30], and hence this topic deserves further attention. As the proper establishment of DNA methylation marks is essential for normal gene expression, aberrant DNA methylation could be responsible for many gene expression abnormalities seen in spermatogenesis. Several studies have analyzed associations between abnormal DNA methylation at certain genes with abnormal sperm parameters (i.e., count, concentration morphology, and/or motility) [7,31]. 

However, these traditional parameters of semen quality are normal in about 15% of infertile males [32]. Our repertoire of tests for semen analysis needs to be expanded to explain these cases. The DNA fragmentation index (DFI) is a measure of the integrity of sperm DNA, which has shown to be a valid predictor of male infertility impacting fertilization and post-embryonic development [32,33,34]. Importantly, perturbations in DNA methylation of specific transcriptional start sites are associated with DFI in human and boar sperm cells [35,36]. Particularly, aberrant methylations of gene promoters corresponding to imprinting, spermatogenesis, and antioxidant systems are seen in infertile males with impaired DNA integrity [36]. These findings suggest that damage-prone DNA regions are more susceptible to DNA methylation alterations, which may lead to abnormal expression of imprinting and non-imprinting genes. 

Methylation abnormalities of methylenetetrahydrofolate reductase (*MTHFR*) have been studied in greater detail than other genes [37,38,39]. MTHFR is fundamental in regulating DNA and folate synthesis, and DNA methylation [40]. Inactivation of MTHFR in mice leads to the hypomethylation of sperm DNA and arrest of spermatogenesis [41]. To explain this, mutations in the *MTHFR* gene reduce the activity of the MTHFR enzyme, decreasing methionine availability and DNA methylation [42,43,44]. Other than mutations, hypermethylation of the *MTHFR* gene could decrease its activity and thereby impair normal methylation of sperm DNA. In this context, males with non-obstructive azoospermia, oligoasthenospermia, and idiopathic infertility can have hypermethylation of the *MTHFR* gene in the testes [45,46,47,48]. Abnormal sperm parameters including concentration, motility, and morphology have also been associated with *MTHFR* hypermethylation in males from infertile couples affected by recurrent spontaneous abortion [31]. Furthermore, hypermethylation of the paternally imprinted gene *H19* has been linked with promoter hypermethylation of the *MTHFR* gene in sperm DNAs from infertile males [31]. 

Other genes have not been as thoroughly investigated, even though some of them play a pivotal role in sperm DNA methylation. For example, DNMTs facilitate de novo methylation but are targets of aberrant methylation or inactivating mutations themselves [49]. Decreased testicular expression of *DNMT1*, *DNMT3A*, and *DNMT3B* and changes in DNA methylation have been found in patients affected with non-obstructive azoospermia [49]. Males who carry the rs4804490 single nucleotide polymorphism (SNP) in the *DNMT1* gene can be at higher risk of idiopathic infertility [50]. Furthermore, infertile males affected with oligozoospermia can have SNPs of *ubiquitin-like containing PHD and RING finger domains 1 (UHRF1)*, an important gene for maintaining proper methylation of DNA throughout spermatogenesis [51,52]. Genes encoding the TET enzymes can also be targets of aberrant methylation. Reduced levels of *TET1*, *TET2*, and *TET3* mRNAs have been found in semen samples from patients affected with oligozoospermia and asthenozoospermia [53]. TET1-deficient mice display a progressive reduction in spermatogonia count and premature reproductive aging by downregulating genes involved in germ-cell differentiation, meiosis, and reproduction [54]. 

Imprinted genes play a critical role in spermatogenesis, and errors in their methylation can impede normal spermatogenesis [55]. Sperm samples from infertile males with abnormal sperm parameters have been found to have disruptions in the methylation of imprinted genes like *MEST* and *H19* [56,57], although their prevalence may have been overestimated due to somatic DNA contamination and genetic variation [58]. Hypermethylation of *MEST* has been associated with male infertility, decreased bi-testicular volume, increased follicle-stimulating hormone (FSH), and abnormal sperm parameters [59,60,61]. Hypomethylation of *H19* has been associated with oligozoospermic, asthenozoospermic, and teratozoospermic infertile males [56,57]. Notably, *H19* hypermethylation has been associated with smoking [62]. On this basis, the methylations status of the ICR of the *H19* gene has been suggested as an epigenetic biomarker of fertility in men with sperm abnormalities [63]. Sperm DNA from infertile males can also have methylation defects at *H19* at the regulatory region CTCF-binding site 6 (CTCF6) located within the DMR of *IGF2-H19* [64]. Consequently, irregular methylation at the *IGF2-H19* CTCF region can inactivate IGF2, which could have a detrimental effect on the development of the embryo and possibly pregnancy outcome [64,65]. Notably, offspring conceived by assisted reproductive technologies (ART) also have been found to have irregular methylation of *IGF2-H19* [66,67] (see section “DNA Methylation Alterations and Artificial Reproductive Technology”).

## 6. DNA Methylation and Female Infertility 

### 6.1. Oogenesis 

The beginning of the ovarian cycle marks the start of the establishment of the DNA methylome specifically in the cohort of oocytes recruited for maturation, which proceeds from the primary to the preantral and antral stages when methylation is completed [14]. Through development, DNA methylation patterns are established in a transcription-dependent manner, leaving non-transcribed genes and intergenic areas hypomethylated, resulting in a DNA methylome that appears bimodal—i.e., composed of hyper- and hypomethylated domains [68].

DNMT1 and DNMT3A/B/L, respectively, are essential for the preservation and establishment of DNA methylation patterns throughout oogenesis. *DNMT3A/B/L* expression levels increase as oocyte development progresses, peaking towards the germinal vesicle stage when de novo methylation is complete, and decreasing after the oocyte reaches the metaphase II stage [68]. The timing of DNA methylation of specific genes is determined by the relative expression of that gene’s local chromatin condition such as histone PTMs and nucleosome density rather than the underlying nucleotide sequence [27,69]. Indeed, genes with high accessibility at transcriptional start sites are linked to greater transcription and, consequently, acquire methylation earlier during oocyte development [70].

DNA methylation preferentially occurs at imprinting control regions (ICRs) and some non-imprinted gDMRs. Imprinted genes have persistent and heritable monoallelic parent-of-origin-specific gene expression that can last a lifetime. Some gDMRs, on the other hand, exhibit transitory or tissue-specific inheritance after fertilization. DNA methylation appears to be unnecessary for oocyte maturation and competence but is required for genomic imprinting and embryo development. Aberrant DNA methylation and failure of genetic imprinting lead to embryonic lethality or congenital diseases such as Beckwith-Weidmann, Angelman, and Prader–Willi syndromes [68]. Oocyte methylome disruptions may also result in developmental problems unrelated to the consequences of perturbed imprinting, such as maternal-to-zygotic transcriptional transition or ovulation failure [24,71,72,73]. 

### 6.2. Oocyte Activation Deficiency 

Following the fusion of sperm and oocyte membranes, the crucial trigger mediating downstream events is oocyte activation, which includes cortical granule exocytosis, resumption of meiosis II, and the induction of early embryogenesis [14,74,75,76]. Calcium (Ca^2+^) oscillations are the key events regulating oocyte activation [77]. Oocyte-activation deficiency (OAD) is a cause of failed ICSI (termed total fertilization failure) that has been thought to arise from male factor infertility but can also involve oocyte-borne causes [74,75,78]. The profile of Ca^2+^ oscillations (i.e., amplitude, wavelength, and duration) is crucial to the successful completion of the immediate post-fertilization events [79,80]; abnormal Ca^2+^ oscillations can impair both implantation and post-implantation development [81]. To relate these findings to DNA methylation, an analysis of oocytes in metaphase II displaying total fertilization failure exhibit significant differences in gene expression compared to healthy controls, with genes involved in meiosis, cell growth, and apoptosis differentially affected [81]. We and others have hypothesized that DNA methylation abnormalities may underlie OAD and explain the deleterious consequences of abnormal Ca^2+^ oscillations on gene expression and post-fertilization development [14,82], but this remains to be tested. 

### 6.3. DNA Methylation and Fertility-Related Diseases

Many studies have now detailed the role of DNA methylation alterations in the pathogenesis of common female comorbidities that negatively impact reproductive potential. These diseases include endometriosis, PCOS, and obesity [83]. 

Endometriosis, a leading global cause of pelvic pain and infertility, has recently been associated with differential methylation profiles of endometrial tissue that distinguishes cases from controls. Several candidate genes (e.g., *HOX-A10*, *PR*, *ESR1*, *CYP19*, *SF-1*, *COX-2*, and *DNMTs*) associated with steroid hormone signaling and DNA methylation have been associated with a significantly higher risk of endometriosis in single studies [84,85,86,87,88,89,90]. A recent study used quantitative trait locus (QTL) mapping to demonstrate 51 loci associated with risk of endometriosis [91]. A recent meta-analysis of PCOS patients demonstrated genome-wide hypomethylation in multiple tissues [91]. Specific genes disproportionately affected by methylation abnormalities in the PCOS phenotype include *FKBP5*, *YAP1*, *CYP19A1*, and *LHCGR* [91]. However, the phenotypic manifestations of women harboring such abnormalities—i.e., whether they demonstrated oligo- or amenorrhea, hyperandrogenism, and infertility—could not be determined [83]. 

Obesity is an important comorbidity associated with female infertility and is associated with a myriad of epigenetic alterations including aberrant methylation in multiple tissues [92]. Female C57BL/6 mice fed a high-fat diet demonstrated global DNA hypomethylation in the ovary [93], albeit the downstream effects of this remain undetermined. Mothers who are overweight/obese pre-pregnancy exhibit differential methylation of 481 CpG sites in cord blood, of which 123 have been related to childhood overweight/obesity [92]. Importantly, cord blood DNA methylation patterns of 14 CpG sites in overweight/obese mothers were statistically significantly associated with child overweight/obesity risk, corresponding to genes involved in energy balance, metabolism, and adulthood metabolic syndrome [92]. Hence, aberrant DNA methylation can be a mechanism of transgenerational risk of adverse health outcomes. 

## 7. DNA Methylation and Age-Related Infertility

Parental age at conception is increasing worldwide but especially in developed nations [94,95]. Both paternal and maternal age have been associated with lower reproductive potential, infertility, poor pregnancy outcomes, higher health risks to the fetus, and higher rates of failure with ART [96,97,98,99,100,101]. This problem is amplified in females suffering from premature ovarian insufficiency. The discussion herein aims to explain these findings through the lens of DNA methylation. 

Epigenetic clocks measure the methylation status of select CpGs in blood or tissue samples to achieve astonishingly accurate predictions of chronological and/or phenotypic age [102]. Notably, epigenetic clocks can identify individuals whose biological age exceeds their chronological age, a concept known as epigenetic age acceleration (EAA) [102]. The most widely utilized clocks in preclinical and clinical studies include the Horvath clock, Hannum clock, PhenoAge, and GrimAge. The Horvath and Hannum clocks provide a strikingly accurate estimate of an individual’s chronological age, GrimAge can estimate lifespan, and PhenoAge can accurately predict the risk of age-related diseases like cancer and Alzheimer’s disease [103,104,105,106]. Other methylation-based aging markers include the blood-based biomarkers Dunedin Pace of Aging methylation (DunedinPOAM) and DunedinPACE [107,108]. Tissue-specific (e.g., brain or cardiac) clocks have also been devised [109,110]. Several studies discussed below have applied the concept of epigenetic aging to determine the causes of age-related decreases in fertility in males and females. However, it should be mentioned that although epigenetic clocks offer a measure of biological age, they are still a way off from being considered ideal ‘gerodiagnostics’ biomarkers, which must fulfill a set of criteria like being responsive to geroscience interventions, acting as a guide for selecting the best geroscience intervention, and their levels in the appropriate body fluid compartment being predictive of a change in clinical state. Various composite gerodiagnostics scores are being developed in therapeutic geroscience clinical trials and may be applicable to age-related infertility in the future. 

### 7.1. Male Infertility

Decreased fertility with increasing paternal age is manifested in abnormal sperm parameters/poor sperm quality, but the precise mechanism remains investigational. By examining genome-wide methylation patterns of sperm DNA from samples collected from 47 couples seeking infertility treatment, Oluwayiose et al. [111] showed significant DNA methylation alterations at 1698 CpGs and 1146 regions with aging, which included a significant number of genes involved in embryonic development. Importantly, differential methylation of four candidate genes, *DEFB126*, *TPI1P3*, *PLCH2*, and *DLGAP2*, was identified as the cause of declining fertility with advancing paternal age in 64% of cases. Applying a machine learning algorithm to analyze sperm DNA methylation patterns from 379 semen samples, Pilsner et al. [112] developed and externally validated a sperm epigenetic aging clock (SEA) to demonstrate that an advanced SEA was associated with longer time-to-pregnancy and shorter gestational ages. Importantly, smokers showed a significantly more advanced SEA compared to non-smokers.

### 7.2. Female Infertility

Age is a principal factor affecting female fertility, with women experiencing a decline in their oocyte reserve and reproductive potential as they get older. Biologically, this may be driven by epigenetic changes. For instance, older women with a natural decline in ovarian function can show DNA methylation abnormalities that result in significantly lower gene expression than young women [113]. Accordingly, ovarian granulosa cells of aged women demonstrate a global decrease in gene transcripts compared to their young counterparts, associated with an increase in DNA methylation marks [113]. Marshall et al. [114] showed that *DMNT1* expression increases in germinal vesicle oocytes with aging, corresponding to an increase in global DNA methylation levels. Another study on aged rats demonstrated the upregulation of DNMT3A and DNMT3B in oocytes and hypermethylation with silencing of autophagy-related genes [115]. Given that impaired autophagy is a biological hallmark of aging [116], it could be the case that age-associated methylation can drive this process in aged oocytes. 

Epigenetic modifications like DNA methylation play a key role in determining the endowment of the initial pool of primordial follicles, hence the notion of epigenetic regulation of primary ovarian insufficiency. Liu et al. [117] reported that methylation levels in oocytes and granulosa cells decreased with oocyte development from the primary to secondary follicles, but then increased in the latter in the tertiary follicle stage. In granulosa cells expressing apoptotic marker TUNEL, DNA methylation levels were decreased during the tertiary follicular stage, suggesting that aberrant DNA methylation induces granulosa cell apoptosis in the follicular stage and may impair oocyte reserve, thereby contributing to premature ovarian insufficiency [117]. In Turner syndrome, a genetic disorder of X monosomy that leads to premature ovarian insufficiency, widespread DNA hypomethylation and differential gene expression have been reported [118,119]. In this regard, fibroblasts of 45, XO individuals demonstrate significant changes in methylation of autosomal genes, including those involved in ovarian function [120]. From blood samples, differentially methylated genes in Turner syndrome include *KDM6A* (involved in germ-cell development) [119]; *USP9X* (involved in oogenesis) [121,122]; *ZFX* (involved in the endowment of the initial germ-cell pool) [123,124]; and potentially *BMP15*, involved in follicular development, polymorphisms of which can be found in women with premature ovarian insufficiency [125,126,127,128,129,130]. However, the methylome of oocytes in Turner syndrome and its consequences on ovarian function is yet to be profiled. 

Hanson et al. [131] showed that women with poor response to ovarian stimulation showed EAA measured by the Horvath clock in their DNAm age in cumulus cells. Building on these findings, Lee et al. [107,132] utilized DunedinPOAM to demonstrate EAA in ART mothers and those with tubal factor infertility, ovulation factor infertility, and unexplained infertility compared to non-ART mothers. It has also been shown that pre-eclamptic pregnancies, which pose a great health risk to both the mother and fetus, are associated with EAA and an increase in burden of cellular senescence, another marker of aging [133]. Devising methods to measure DNAm Age specifically in the ovary through a tissue-specific clock would be the next step in furthering these observations. 

## 8. DNA Methylation Alterations in Assisted Reproductive Technology

Animal studies have shown that ART can alter normal DNA methylation of imprinting-associated genes [134]. Gomes et al. [135] discovered that abnormal methylation at the *KvDMR1* ICR was present in children born via ART, suggesting that the establishment and maintenance of genomic imprinting may be affected by ART. Oocyte vitrification, a popular technique in the field of ART, can alter the DNA methylation profiles of oocytes [136]. In humans, there is evidence of a plausible but not proven association between ART and imprinting disorders like Beckwith–Wiedemann syndrome, Angelman syndrome, and Silver–Russell syndrome [134,137]. However, it is important to mention that studies have shown no significant association between ART and the risk of imprinting disorders [138,139,140,141]. For instance, comparing the methylome of ICSI and naturally conceived children revealed significant DNA methylation differences but with a small effect size [142]. These small epigenetic differences were found to resolve during adulthood with no impact on development and health [138]. Together, these findings do indicate potential DNA methylation in ART children but no causal association with any impact on health or disease susceptibility. 

## 9. Conclusions 

This review provided a concise overview of the involvement of DNA methylation in regulating sperm and oocyte features pertinent to infertility. Recent studies suggest that DNA methylation alterations in sperm as a cause of infertility might have been overestimated, and that the functional impact of differential methylation because of ART on fetal health and development is not significant. Although sperm methylome has been studied for years, only a handful of genes have been focused on. Additionally, the list of modifiable risk factors that impact fertility through DNA methylation in both males and females is limited to smoking and emerging studies on obesity. The impact of age on epigenetic changes like DNA methylation needs to be reflected in stratifying comparisons of the epigenome between infertile and fertile individuals according to age. As has been done in the case of sperm, developing an ovary-specific epigenetic clock can provide insight into the epigenomic alterations in ovaries with aging and the impact of external factors like physical fitness, smoking, obesity, and comorbidities. Lastly, the impact of DNA methylation as a cause and consequence of abnormal Ca^2+^ oscillations during oocyte activation remains investigational. Future studies investigating these uncertainties and addressing caveats of current data may place DNA methylation as a clinical biomarker of infertility or a therapeutic target.
